# Nox4 Mediates Renal Cell Carcinoma Cell Invasion through Hypoxia-Induced Interleukin 6- and 8- Production

**DOI:** 10.1371/journal.pone.0030712

**Published:** 2012-01-27

**Authors:** John P. Fitzgerald, Bijaya Nayak, Karthigayan Shanmugasundaram, William Friedrichs, Sunil Sudarshan, Assaad A. Eid, Thomas DeNapoli, Dipen J. Parekh, Yves Gorin, Karen Block

**Affiliations:** 1 Audie L. Murphy Memorial Hospital Division, South Texas Veterans Health Care System, University of Texas Health Science Center, San Antonio, Texas, United States of America; 2 Division of Nephrology, Department of Medicine, University of Texas Health Science Center, San Antonio, Texas, United States of America; 3 Department of Urology, University of Texas Health Science Center, San Antonio, Texas, United States of America; 4 Department of Pathology, University of Texas Health Science Center, San Antonio, Texas, United States of America; Vanderbilt University Medical Center, United States of America

## Abstract

**Background:**

Inflammatory cytokines are detected in the plasma of patients with renal cell carcinoma (RCC) and are associated with poor prognosis. However, the primary cell type involved in producing inflammatory cytokines and the biological significance in RCC remain unknown. Inflammation is associated with oxidative stress, upregulation of hypoxia inducible factor 1-alpha, and production of pro-inflammatory gene products. Solid tumors are often heterogeneous in oxygen tension together suggesting that hypoxia may play a role in inflammatory processes in RCC. Epithelial cells have been implicated in cytokine release, although the stimuli to release and molecular mechanisms by which they are released remain unclear. AMP-activated protein kinase (AMPK) is a highly conserved sensor of cellular energy status and a role for AMPK in the regulation of cell inflammatory processes has recently been demonstrated.

**Methods and Principal Findings:**

We have identified for the first time that interleukin-6 and interleukin-8 (IL-6 and IL-8) are secreted solely from RCC cells exposed to hypoxia. Furthermore, we demonstrate that the NADPH oxidase isoform, Nox4, play a key role in hypoxia-induced IL-6 and IL-8 production in RCC. Finally, we have characterized that enhanced levels of IL-6 and IL-8 result in RCC cell invasion and that activation of AMPK reduces Nox4 expression, IL-6 and IL-8 production, and RCC cell invasion.

**Conclusions/Significance:**

Together, our data identify novel mechanisms by which AMPK and Nox4 may be linked to inflammation-induced RCC metastasis and that pharmacological activation of AMPK and/or antioxidants targeting Nox4 may represent a relevant therapeutic intervention to reduce IL-6- and IL-8-induced inflammation and cell invasion in RCC.

## Introduction

Inflammation is associated with cancer, including renal cell carcinoma (RCC) [1], [2]. Inflammation is caused by a variety of pathogenic and environmental factors. Various pathogenic and environmental factors cause inflammation, which involve oxidative stress, upregulation of hypoxia inducible factor 1-alpha, and production of pro-inflammatory gene products, together suggesting that hypoxia may play a role in inflammatory processes in RCC [3]. Cytokines have been detected in the plasma of patients with advanced renal cancer and is associated with poor outcomes, but the source of cytokine production and the biological significance of cytokine secretion in RCC are not known [4]. Primary cells involved in the release of cytokines include lymphocytes and macrophages; however, macrophage infiltration is not a predominant feature of RCC [5]. Epithelial cells have also been implicated in cytokine release, although the stimuli to release and molecular mechanisms by which they are released remain unclear.

Reactive oxygen species (ROS) have been implicated in inflammatory pathways and cancer through regulation of the redox state of the target cells. In the kidney, ROS are primarily produced by NAD(P)H oxidases of the Nox family. Our laboratory and others has identified Nox oxidases, and particularly the isoform Nox4, as a major source of ROS in renal cell carcinoma [6], [7], [8] and our group has previously demonstrated that AMP-activated protein kinase (AMPK) is a novel upstream regulator of Nox oxidase protein expression and activity [9], [10], [11]. AMPK is a highly conserved sensor of cellular energy status and a role for AMPK in the regulation of cell inflammatory processes has recently been demonstrated [12], [13], [14]. Pharmacological activation of AMPK, reduces tumor growth *in vitro* and in a xenograft mouse model [15], [16], but the role of AMPK in inflammatory processes in RCC has not been studied.

Metastatic renal cancers are commonly refractory to current therapy. Molecular identification of signals that elicit RCC metastasis and the pathways that sense and propagate the response to invade are lacking. The objectives of this study were to identify 1) renal epithelial-dependent nonpathogen-derived-pro-inflammatory gene products in RCC 2) characterize the microenvironment and signaling pathways involved in pro-inflammatory release and 3) determine the cellular mechanisms and biological consequences of cytokine secretion in RCC. Here, we describe a novel interplay between hypoxia, Nox4 and AMPK that modulates inflammatory processes in RCC.

## Materials and Methods

### Materials

N-Acetyl-L-cysteine (NAC, 10mM) and diphenyleneiodonium chloride (DPI, 5uM) and 5-aminoimidazole-4-carboxamide-1-riboside (AICAR: 1mM) were purchased from Sigma.

### Cell Lines and Cultures

Established (American Type Culture Collection) human renal proximal tubular cells, HK2; and VHL-deficient cells, RCC 786-O and RCC4, were maintained in RPMI (HK2) or Dulbecco's modified Eagle's medium (RCC 786-O, RCC4) (DMEM, Invitrogen) supplemented with 10% fetal bovine serum, 2 mM L-glutamine, 50 units/ml of penicillin and 50 µg/ml streptomycin sulfate at 37°C with 5% CO2. *Ex vivo* RCC primary cells were established from a histologically identified clear cell tumor. The tumor mass was obtained at the time of tumor resection and processed under sterile conditions: washed with ice cold PBS, minced and trypsinized to obtain a single cell suspension and maintained in DMEM-complete media as used for established cultured RCC cells. Cells were maintained at 37°C with 5.0% CO_2_ and grown to ∼65% confluency prior to experimental procedures.

### Western Blotting Analysis

Immunoblotting was performed by running typically between 25–70 µg total protein and incubated with the indicated primary antibodies: Rabbit polyclonal Nox4 antibody (1∶1000) and GAPDH (1∶4000) was purchased from Santa Cruz. Total MAPK (1∶1000), was purchased from cell signaling. The immunoblots were washed and incubated with Goat anti-rabbit-coupled horseradish peroxidase (Biorad) followed by chemiluminescence using ECL reagent (Amersham-Parmacia).

### Induction of hypoxia

All cells were cultured in serum-free media ∼18 hrs prior to hypoxia exposure for indicated times. Cultured cells were placed in a modular incubator chamber (Don Whitley Scientific Limited, MACS VA500) that was flushed with a gas mixture (0.5% O2 5.0% CO2 94.5% N2) to remove atmospheric air. Within 30 minutes, the desired level of hypoxia was achieved. Samples of media were analyzed for pH at each experimental time interval (0–48 hours). All buffers and reagents used were allowed to equilibrate for at least 6 hours in the hypoxic chamber and hypoxia-induced conditioned media were harvested or cell lysates prepared in the hypoxic chamber on ice prior to removal.

### Cell viability

Cell viability was determined by trypan blue exclusion. After hypoxic incubation for 72 h, % cells were assayed for cell viability using dye exclusion. Live cells with intact cell membranes exclude trypan blue whereas dead cells do not. Hypoxic cell suspension was mixed with 0.4% trypan blue in PBS and allowed to sit at room temperature for 3 minutes and placed in a hemocytometer and counted under a binocular microscope. The number of unstained (viable) and stained (nonviable) was counted. All experiments were carried out in triplicates.

### Transfection

A SMARTpool consisting of four short or small interfering RNAs (siRNA) duplexes specific for human Nox4 was obtained from Dharmacon. The SMARTpool of siRNA for Nox4 was transfected using the Amaxa system. Briefly, RCC 786-O cells were plated in complete medium to obtain 65% confluence on the day of transfection. 400nM of scrambled control or smart pool siRNA Nox4 were added to the cells and transfected using the AMAXA system as to the manufactures specifications. After transfection, cells were gently plated in complete media overnight and subjected to serum starvation prior to being placed in the hypoxic chamber.

### ELISA analysis

Established cultured normal proximal tubular epithelial cells (HK2), VHL-deficient renal carcinoma cells (RCC 786-O and RCC4), or primary RCC cultured *ex vivo* cells, were plated on a 100 mm plate, serum starved over night and exposed, or not, to hypoxia as described above. Inflammatory cytokines were measured using the Mosaic™ ELISA (R&D systems) and the IL-6 and IL-8 ELISA (R&D systems) for specific cytokines. All assays were preformed as to the manufactures specifications.

### *In vitro* matrigel invasion assays

Invasion of tumor cells were evaluated using a Matrigel coated modified Boyden chamber (Biocoat ^TM^ Matrigel^TM^ Invasion Chamber; Becton Dickinson GmbH, Heidelberg, Germany) as per manufactures instruction. Briefly, 30,000 were seeded into the upper well of the chamber containing serum free media. The lower well contained culture medium containing 10% FBS as a chemoattractant. After 24 hours the excess cells were wiped away and the chambers stained for invaded cells. At 24 hrs, the loose cells on the top of the matrigel membrane were removed with a cotton swab. The migrated cells were washed with PBS and fixed with 70% ethanol. Staining was preformed using 0.1% crystal violet to visualize the migrated cells. The transmigrated cells in the membrane were counted using a light microscope at 10X magnification. For each experiment, 10 random high power fields were photographed, digitalized and counted. *In vitro matrigel invasion using recombinant IL-6 and IL-8*. Invasion chambers were prepared as above. Purified IL-6 (10ng/ml) and IL-8 (15ng/ml) were added at the indicated concentrations to the top chamber. Results were recorded as specified. *In vitro matrigel invasion using conditioned media*. Invasion chambers were prepared as above. Condition media from 786-O cells that were exposed to 48 hours of hypoxia, 786-0 cells with a stable Nox4 knock down exposed to 48 hours of hypoxia and 786–0 cells treated with AICAR and exposed to 48 hours of hypoxia was added to the upper chamber of the invasion assay. Results were recorded as above. *In vitro matrigel invasion using cultured primary cells*. Primary culture cells were maintained at normoxia and kept in complete media with or without 24 hour of AICAR pretreatment. These were then added to the upper chamber of the Matrigel invasion assay. Results were collected as above.

### Human Tumor Specimens/Ethics Statement

Tumor samples and normal corresponding tissue from patients with RCC were obtained from the Department of Urology at the University of Texas Health Science Center at San Antonio under approved IRB protocol HSC20070777N. This is a “non-human/nonresearch” protocol, which collects unwanted tissue. The tumors for this study were histologically classified as clear cell renal carcinoma and staged according to the TNM classification. The collection, handling and non-identifiers of human samples was carried out according to a protocol approved by the University of Texas Health Science Center at San Antonio, Institutional Review Board.

## Results

### Hypoxia-induces interleukin-6 and 8-production solely in RCC cells

**Figure 1:**
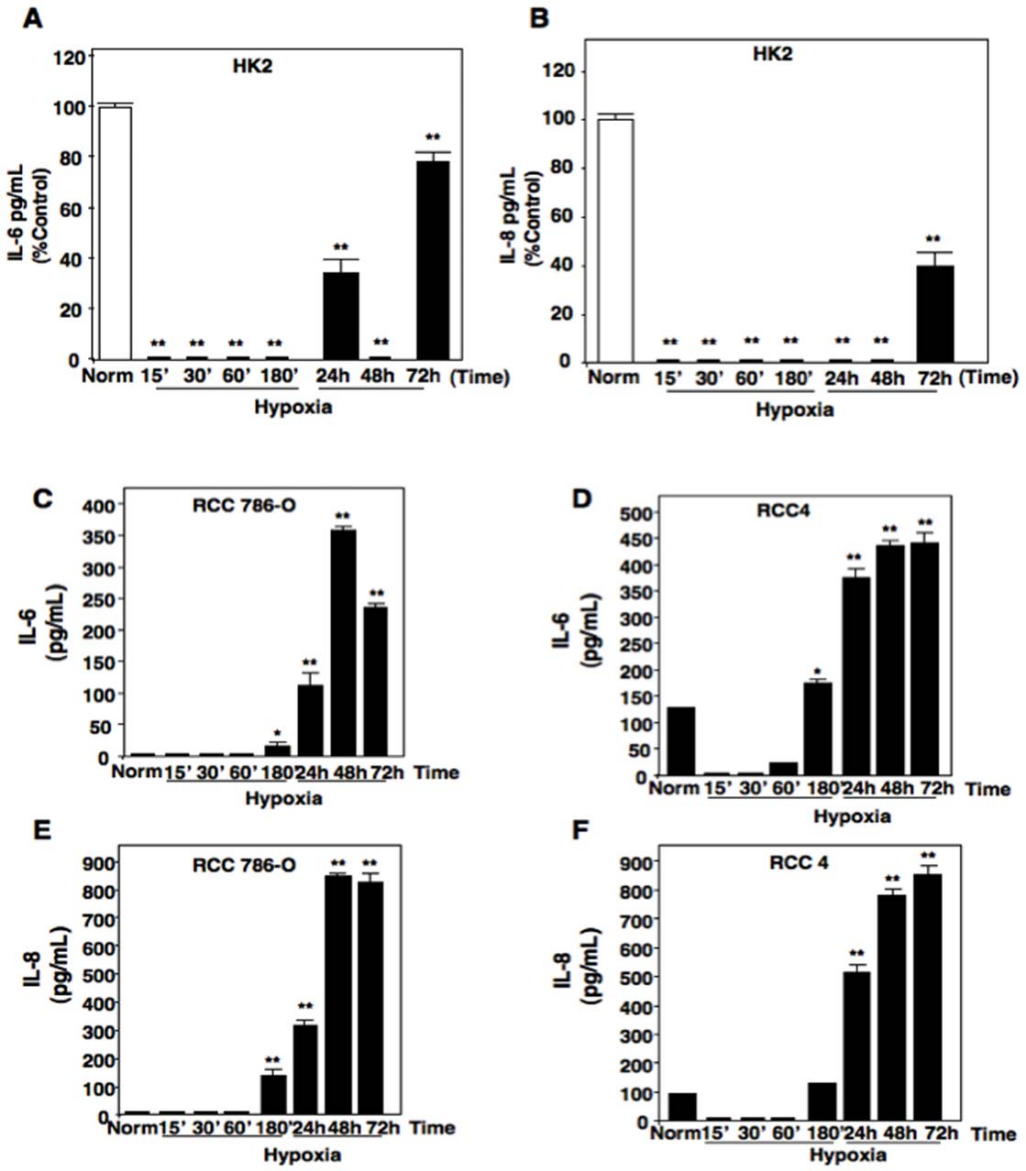
Hypoxia induces IL-6 and IL-8 production solely in RCC cells. A) Interleukin-6 (IL-6) and B) Interleukin-8 (IL-8) secretion by normal proximal tubular epithelial cells (HK2) exposed to normoxic (norm) or hypoxic conditions for short (T15-180min) or long (T24-T72hr) time points was determined by ELISA. C, D) IL-6 and E, F) IL-8 secretion by renal carcinoma cells (RCC 786-O and RCC4) exposed to normoxic (norm) or hypoxic conditions for short (T15-180min) or long (T24-T72hr) time points was determined by ELISA as outlined in materials and methods.

Cytokines have been detected in the plasma of patients with advanced renal cell carcinoma (RCC) and poor prognosis; however, macrophage infiltration is not consistently detected in RCC tumors [4], [5]. Tumors are often heterogenous in oxygen tension [17]. Taken together, we sought to identify the cell type and stimulus that is responsible for cytokine production in RCC. Cultured normal proximal tubular epithelial cells (HK2) or VHL-deficient RCC (RCC 786-O and RCC4) cells were subjected to time controlled normoxic (21% oxygen) or hypoxic (0.5% oxygen) conditions for short (T15′-180′) or long (T24hr-72 hrs) times. Hypoxic exposure did not alter cell viability or pH in our studies (data not shown). Cell media was harvested under normoxic conditions or in the hypoxic chamber. Mosaic Elisa analysis was conducted for detection of cytokines and interleukins including, CD40-L, INF-gamma, interleukins- 1A, -1B, -6, -8, and -17, and TNF-alpha. At normoxic conditions, HK2 cells expressed minor amounts of interleukins-6 (Fig. 1A) and -8 (Fig. 1B) whereas other cytokines on the panel were barely detected (data not shown). Importantly, when HK2 cells were subjected to hypoxic conditions, production of interleukins-6 and -8 (IL-6 and IL-8) were immediately and significantly reduced. Even at later time points when levels of IL-6 and IL-8 were modestly increased, the levels never reached basal starting levels compared to normoxic conditions. On the other hand, VHL-deficient RCC cells exposed to normoxic conditions expressed IL-6 and IL-8 at very low levels (Figs. 1C–F) and unlike HK2 cells, hypoxic conditions resulted in a significant increase of IL-6 and IL-8 production starting at 180′ and steadily increasing to 4–5 fold by 72 hours (Figs. 1 C–F). Other cytokines tested, CD40-L, INF-gamma, interleukins- 1A, -1B, -and -17, and TNF-alpha were not detected under normoxic or hypoxic conditions in RCC 786-O or RCC4 cells (Figs. S1, S2, respectively). Taken together, these results suggest that RCC cells solely express IL-6 and IL-8 under hypoxic conditions.

### Nox4 mediates hypoxic-induced interleukin-6 and 8-production in RCC cells

**Figure 2:**
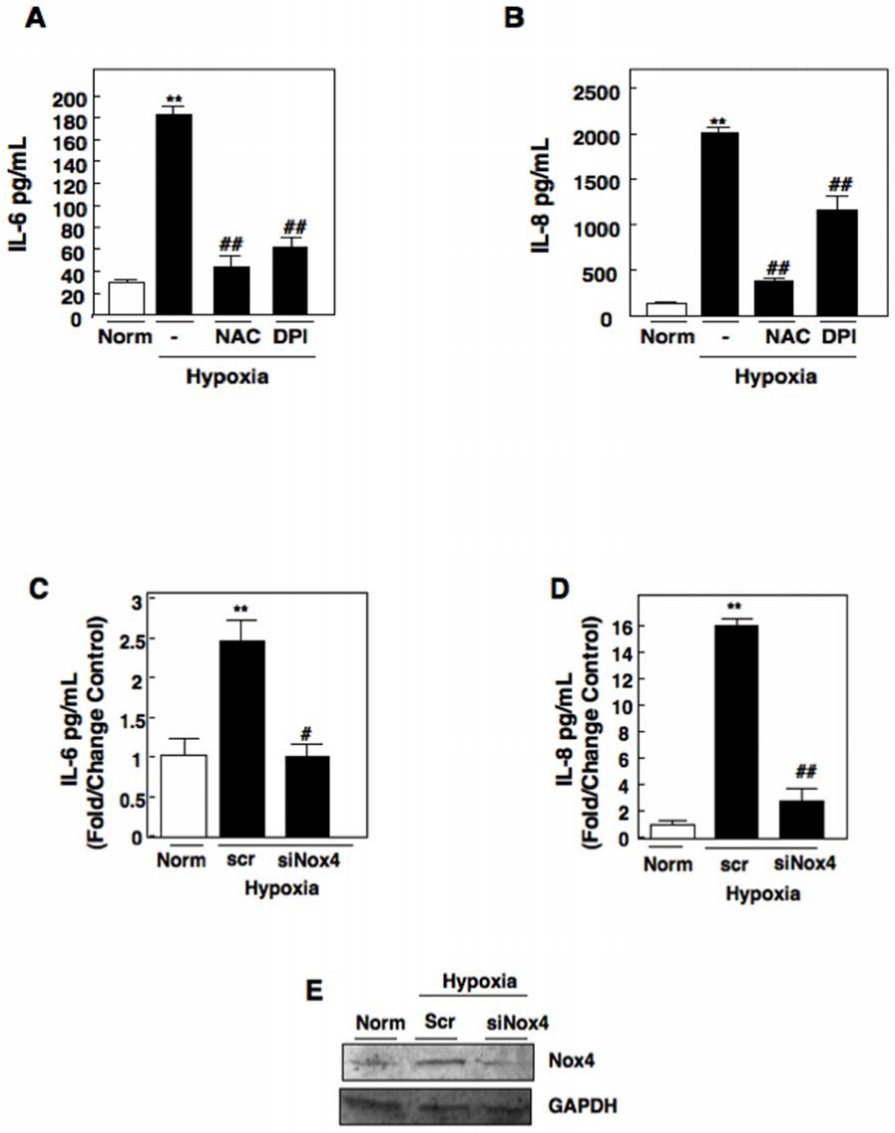
Effects of antioxidants on Hypoxia induced IL-6 and IL-8 production in RCC cells. A) Interleukin-6 (IL-6) and B) Interleukin-8 (IL-8) secretion was measured using ELISA analysis in RCC 786-O cells pretreated for 30 min in buffer alone (-), N-Acetyl-L-cysteine (NAC) or a flavoprotein inhibitor (DPI) and exposed to normoxic (norm) or hypoxic conditions for 48 hrs. The data were quantitated from three independent experiments and the results are expressed as the means + S.E. **, p<0.01 versus norm and #*#*, p<0.01 versus hypoxic control (-). C) IL-6 and D) IL-8 secretion was measured using ELISA analysis in RCC 786-O cells transfected with scrambled control (Scr) or small interfering RNA to Nox4 (siNox4) exposed to normoxic (Norm) or hypoxia for 48 hrs. The data were quantitated from three independent experiments and the results are expressed as the means + S.E. **, p<0.01 versus norm, *#*, p<0.05, and *##*, p<0.01 versus hypoxic scr control. E) RCC 786-O cells transfected with scrambled control (Scr), small interfering RNA to Nox4 from C, D were analyzed by Western blot with Nox4 antibody or GAPDH control.

We next wanted to elucidate the molecular mechanisms by which hypoxia induces IL-6 and IL-8 production in RCC cells. Reactive oxygen species (ROS) have been implicated in cytokine production [18], [19]. Our laboratory has previously demonstrated that VHL-deficient RCC cells demonstrate higher levels of ROS mediated by Nox oxidases [7], [8]. To determine if Nox-dependent ROS mediate hypoxia-induced IL-6 and IL-8 production, we pre-incubated RCC 786-O cells with buffer alone (-), the Nox inhibitor, diphenyleneiodonium (DPI) or the ROS scavenger, N-Acetyl Cysteine (NAC) for 30 min and subjected to hypoxia or normoxia for 48 hours. As previously detected, IL-6 and IL-8 production was significantly enhanced at 48 hours (hypoxia (-)) compared to normoxic control. However, RCC cells pre-incubated with DPI or NAC resulted in a significant reduction of IL-6 and IL-8 production compared to hypoxic controls (-) (Figs. 2A and 2B). Together, these data suggest that Nox-dependent ROS generation are involved in hypoxia-induced IL-6 and IL-8 production in RCC.

Our laboratory has previously demonstrated that the Nox catalytic subunit, Nox4 is highly expressed in VHL-deficient RCC 786-O cells and is a major source of oxidative stress in RCC [7]. To determine if Nox4 was involved in hypoxia-induced IL-6 and IL-8 production, we transfected RCC-786-O cells with small inhibitory RNAs against Nox4 (siNox4) or scrambled control (scr). Transfected cells were exposed to hypoxic conditions for 48 hrs. We found silencing of Nox4 (siNox4) significantly reduced IL-6 and IL-8 production in cells exposed to hypoxic conditions compared to mock (-) or scrambled control (scr) (Fig. 2C & 2D). Confirmation of Nox4 knockdown was performed in parallel using Western blot analysis (Fig. 2E). GAPDH was used as a loading control. Taken together, our data suggest that Nox4 mediates hypoxia-induced IL-6 and IL-8 production in RCC cells.

### Activation of AMPK reduces Nox4-dependent IL-6 and IL-8 production under hypoxic conditions

**Figure 3:**
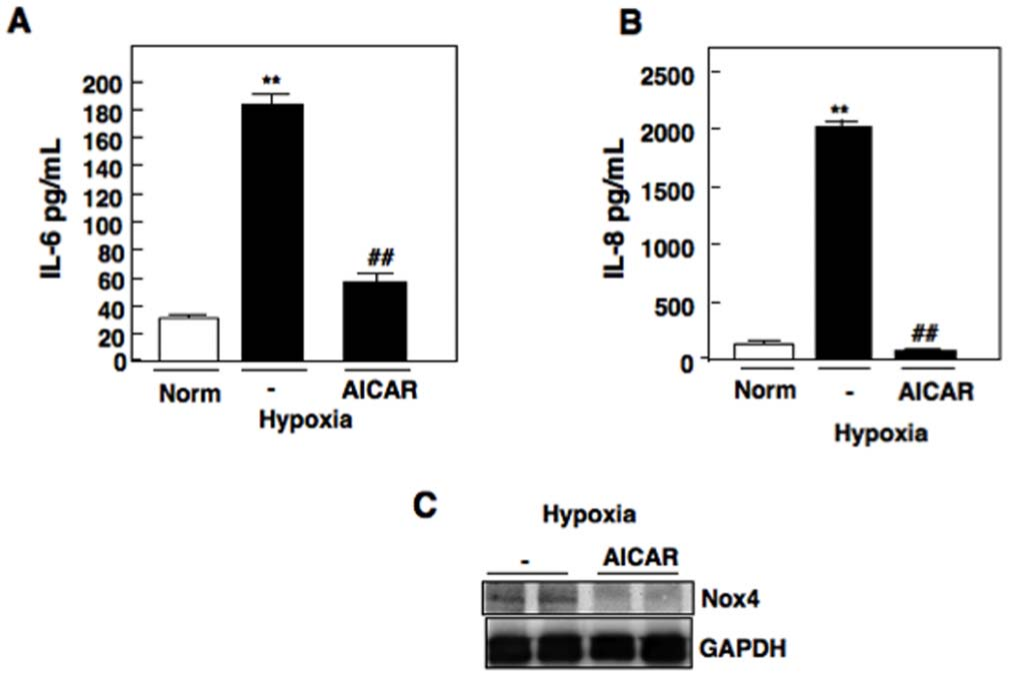
Effects of AMPK activation on Hypoxia induced IL-6 and IL-8 production in RCC cells. A) Interleukin-6 (IL-6) and B) Interleukin-8 (IL-8) secretion was measured using ELISA analysis in RCC 786-O cells pretreated for 30 min in buffer alone (-), or the AMPK activator, aminoimidazole-4-carboxamide-1-riboside 5-aminoimidazole-4-carboxamide-1-riboside (AICAR) and exposed to normoxic (norm) or hypoxic conditions for 48 hrs. The data were quantitated from three independent experiments and the results are expressed as the means + S.E. **, p<0.01 versus norm, *##*, p<0.01 versus hypoxic control (-). C) Cell lysates were prepared from cells treated with AICAR, (Fig. 3 *A, B)* and analyzed for Nox4 expression or GAPDH control by Western blot analysis.

Our group has previously demonstrated that activation of AMPK using 5-aminoimidazole-4-carboxamide riboside (AICAR), a cell permeable drug that is converted to ZMP, an analogue of AMP, reduces Nox4 expression and Nox4-dependent ROS generation [11]. Activation of AMPK has been of recent clinical interest for the treatment of renal cancer. To determine if AMPK is an upstream regulator of Nox4 and mediates hypoxia-induced IL-6 and IL-8 production, we exposed RCC 786-O cells to normoxic, hypoxic, or pre-incubated RCC cells with the AMPK activator, AICAR and subjected the cells to hypoxia for 48 hours. As previously noted, IL-6 and IL-8 production was significantly enhanced at 48 hours (hypoxia (-)) compared to normoxic control. However, RCC cells pre-incubated with AICAR resulted in a significant reduction of IL-6 and IL-8 production compared to hypoxic controls (-) (Figs. 3A and 3B). Importantly, AICAR reduced Nox4 protein expression (Fig. 3C). GAPDH was used as a loading control.

### IL-6 and IL-8 induces RCC cell invasion

Circulating plasma levels of interleukins are associated with poor prognosis. Renal carcinomas are highly metastatic. To elucidate the biological significance of IL-6 and IL-8 production in RCC cells exposed to hypoxia, we examined cell invasion. RCC 786-O cells were plated in a Boyden chamber in the presence of recombinant IL-6, IL-8 or the combination of IL-6 and IL-8 or buffer alone (control). We find that incubation of IL-6 alone increased cell invasion compared to buffer treated media, however, not to significance. Addition of IL-8 resulted in a significant increase of cell migration and addition of IL-6 and IL-8 further increased significant cell migration.

**Figure 4:**
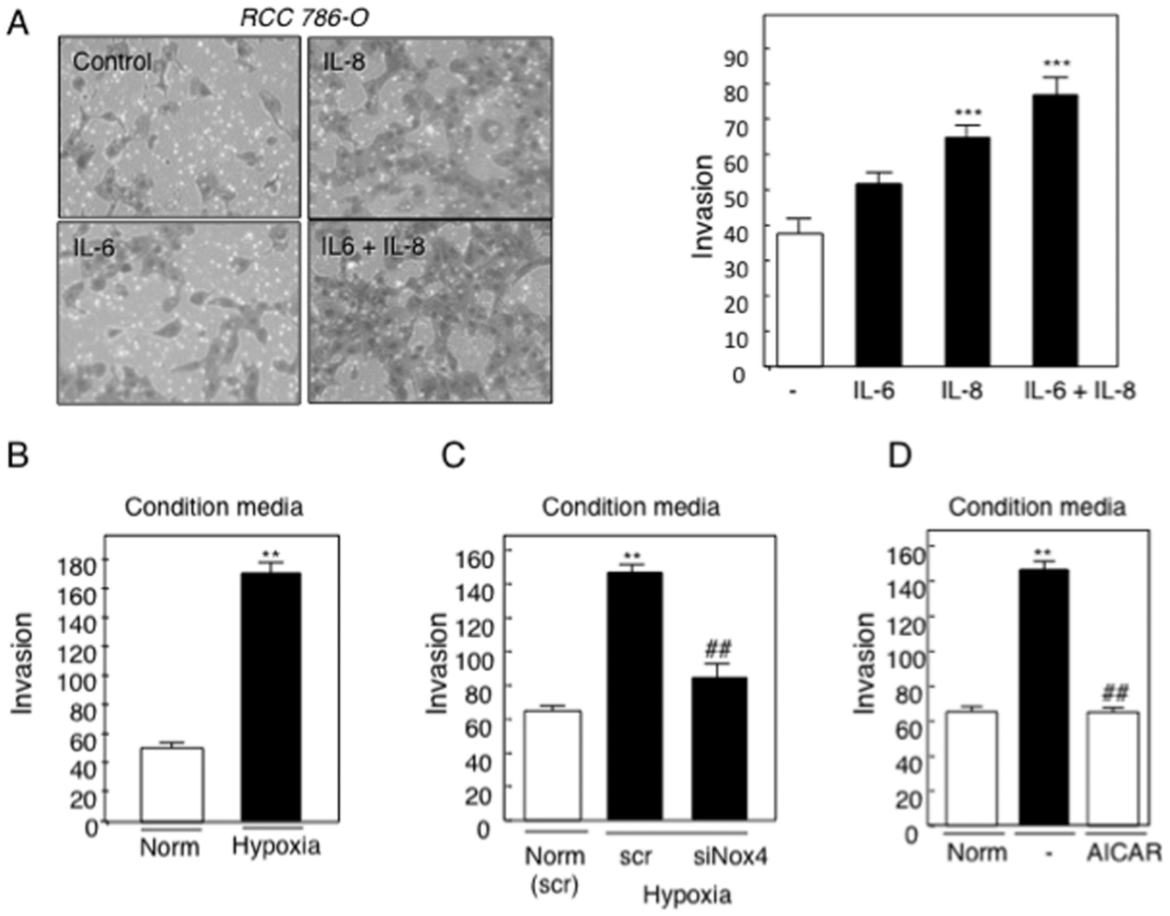
Effects of IL-6 and IL-8 on RCC cell invasion. A) RCC 786-O cells were treated with recombinant IL-6 and/or IL-8 for 18 hours and cell invasion was analyzed by Boyden chamber as described in materials and methods. *Left panel,* the invaded cells were photographed and *right panel,* invaded cells were counted and quantified. The graph is representative of ten independent experiments and the results are expressed as the means + S.E. ***, p<0.001 versus buffer control (-). B) RCC 786-O cells were incubated with condition media from RCC 786-O cells subjected to 48hr norm or hypoxic conditions (Fig. 1C,E) for 18 hours and cell invasion was analyzed by Boyden chamber. C) RCC 786-O cells were incubated with condition media from RCC 786-O cells in normoxic media (norm) or hypoxic media from Fig. 2 D, E transfected with scrambled control (scr) or siRNA for Nox4 (siNox4). D) RCC 786-O cells were incubated with condition media from RCC 786-O cells in normoxic media (norm) or hypoxic media from Fig. 3A,B incubated with buffer control (-) or AICAR. B, C, D, Invaded cells were quantified from three independent experiments and expressed as the means + S.E. **, p<0.01 versus norm, *##*, p<0.01 versus hypoxic control (-).

As conditioned medium (CM) from RCC 786-O cells incubated in hypoxia exhibited high levels of IL-6 and IL-8, which was reduced by siNox4 and AICAR treatment, we examined the ability of CM, subjected to the aforementioned conditions, to induce cell migration. RCC 786-O cells were plated in the Boyden chamber using CM from RCC 786-O cells exposed to normoxic or hypoxic conditions for 48 hours. RCC 786-O cells exposed to hypoxic CM significantly stimulated the migratory activity of RCC 786-O 3 fold compared to normoxic culture conditions (Fig. 4B). We next tested CM from normoxic or hypoxic RCC 786-O cells silenced of Nox4 (siNox4) or scrambled control. CM harvested from hypoxia-induced scrambled transfected cells resulted in a 2.5 fold increase in cell migration compared to normoxic scr control; whereas CM from hypoxia-induced RCC 786-O cells transfected with siRNA Nox4 (siNox4) showed a significant reduction in cell migration compared to hypoxia-induced scr control (Fig. 4C). Similarly, when CM from RCC 786-O cells exposed to hypoxia in the presence of AICAR was added to RCC 786-O cells in the Boyden chamber, hypoxia induced cell migration was reduced back to basal levels (Fig. 4D). Taken together, this data suggests that IL-6 and IL-8 mediate cell invasion in RCC cells.

### AICAR reduces IL-6, IL-8 and cell invasion in *ex-vivo* RCC cells

**Figure 5:**
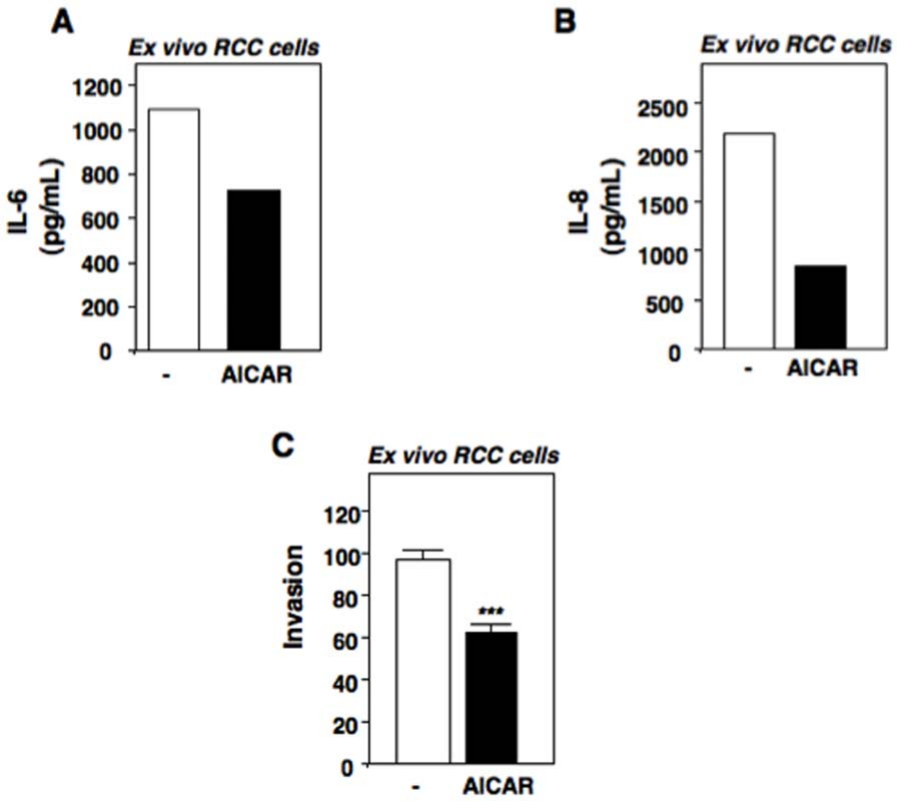
*Ex-vivo* human RCC tumor cells demonstrate reduced cell invasion with AICAR treatment. Human RCC cells were cultured as described in materials and methods. Freshly prepared *ex-vivo* cells were incubated with AICAR for 48 hours and A) Interleukin-6 (IL-6) and B) Interleukin-8 (IL-8) was measured by ELISA analysis. C) Freshly prepared *ex-vivo* human RCC cells were plated in a Boyden chamber in the presence (+) or absence (-) of AICAR for 18 hours. Invaded cells were quantified from three independent experiments and expressed as the means + S.E. ***, p<0.001 versus buffer control (-).

Activation of AMPK by AICAR reduces Nox4 expression and production of IL-6 and IL-8 in cultured RCC cells exposed to hypoxia. Furthermore, independent studies have suggested that activation of AMPK suppresses proinflammatory responses [12], [13], [14]. To determine if AMPK activators are relevant in clinical specimens, we cultured RCC cells from a human tumor of clear cell histology and incubated the cells in the presence or absence of AICAR. Elisa analysis of IL-6 and IL-8 demonstrated a reduction of IL-6 and IL-8 levels in the *ex-vivo* RCC cells exposed to AICAR (Figs. 5A,B). Importantly, cell invasion was also significantly reduced compared to buffer treated controls (Fig. 5C).

## Discussion

We have identified for the first time that interleukin-6 and interleukin-8 (IL-6 and IL-8) are secreted solely from RCC cells exposed to hypoxia. Furthermore, we demonstrate that the NADPH oxidase isoform, Nox4, play a key role in hypoxia-induced IL-6 and IL-8 production in RCC. Finally, we have characterized that enhanced levels of IL-6 and IL-8 result in RCC cell invasion and that activation of AMPK reduces Nox4 expression, IL-6 and IL-8 production, and RCC cell invasion. Together, these data identify novel mechanisms by which Nox4 may be linked to inflammation-induced RCC metastasis.

Interleukins have been detected in the serum of patients with metastatic renal carcinoma; however, the stimulus necessary for interleukin production and cell type important interleukin secretion were previously unknown [4], [20]. Inflammatory cells, which normally secrete interleukins, such as macrophages, are not a predominant feature of RCC [5]. Therefore, we sought other environmental factors that may be involved. Solid tumors exhibit intratumor hypoxic states, where regions of low oxygen (hypoxia) and necrosis are common [17], [21]. Importantly, we find that cultured normal proximal tubular cells exposed to hypoxia resulted in downregulation of IL-6 and IL-8 production whereas cultured VHL-deficient RCC cell lines (RCC 786-O and RCC4) resulted in a significant increase of IL-6 and IL-8 production. Importantly, we do not find that hypoxia induces significant changes in cellular pH or cell death under our experimental conditions (data not shown). Previous studies have demonstrated enhanced production of IL-6 and IL-8 in various cancer cells such as pancreatic, ovarian and glioblastoma exposed to hypoxia, but our study demonstrates for the first time that normal epithelial cells down-regulate whereas predominantly, renal tumor cells upregulate interleukin expression under hypoxic conditions [22], [23], [24], [25]. Additionally, we have identified the signaling pathways that mediate hypoxia-induced IL-6 and IL-8 production in RCC cells.

Hypoxia sensing and related signaling events, including activation of hypoxia-inducible factor 1 (HIF-1), now suggest that the NADPH oxidase subunit, Nox4, serve as oxygen sensors. For example, Nox4 is increased by chronic exposure of mice to hypoxia [26]. Additionally, the human Nox4 promoter harbors a hypoxia responsive element (HRE) by which HIF-1alpha mediates rapid Nox4 induction during hypoxia in pulmonary artery smooth-muscle cells [27]. VHL-deficient RCC 786-O and RCC4 cells express hypoxia-inducible factors -2 alpha (HIF-2alpha) and hypoxia-inducible factors 1- and 2- alpha (HIF-1alpha/HIF-2alpha) respectively. The fact that normal proximal tubular cells, exposed to hypoxia do not induce interleukin expression suggests that Nox-dependent production of IL-6 and IL-8 may not be mediated by hypoxia-inducible factors. In support of this, we find that incubation of RCC cells with the HIF inhibitor; 3-(5′-hydroxymethyl-2′-furyl)-1-benzylindazole (YC-1) did not block IL-6 and IL-8 production in RCC cells exposed to hypoxia (data not shown).

We examined other potential upstream regulators of Nox4. Recently it has been demonstrated that activation of AMPK using AICAR suppresses pro-inflammatory responses; however, the biological significance of AMPK activation in hypoxia-induced interleukin expression and RCC metastasis is unknown. Our group has previously demonstrated that activation of AMPK down regulates Nox4 expression and Nox4-dependent apoptosis in renal podocytes [11]. AMPK is a key nutrient and energy sensor in cells and in general, AMPK is activated under hypoxic conditions. We find that AMPK activity is lower in cultured RCC cells compared to normal renal epithelial cells and that hypoxia does not stimulate AMPK activation in RCC cells (data not shown). Together, this may explain why normal epithelial cells reduce interleukin expression under hypoxic conditions in contrast to RCC cells, which do not exhibit the ability to inhibit interleukin expression under hypoxic conditions. The mechanisms by which AMPK activity are reduced and/or inactivated in RCC are under current investigation. Importantly, however, we do find that pharmacological activation of AMPK in RCC cells using AICAR reduces Nox4 expression and hypoxia-induced IL-6 and IL-8 production. This is the first study demonstrating molecular suppression of pro-inflammatory production by activation of the AMPK pathway. Although we did not examine downstream mediators of Nox4 induced IL-6 and IL-8 production, we are currently examining the role of the redox-sensitive transcription factor, NF-kB, which is potent intracellular regulator of interleukin secretion. Reactive oxygen species have been implicated as second messengers involved in the activation of NF-κB as several studies have demonstrated that activation of NF-κB by nearly all stimuli can be blocked by antioxidants [28], [29], [30] and Nox4 inhibition [31]. Reactive oxygen species on NF-κB activation is further supported by studies demonstrating that hydrogen peroxide induces NF-κB-dependent interleukin-8 expression in endothelial cells, which contributes to the angiogenic phenotype [32]. The role of Nox4-dependent activation of NF-kB in response to hypoxia in RCC is under current investigation.

The biological significance and clinical relevance of IL-6 and IL-8 levels in RCC is presently unknown. We find that RCC cells exposed to IL-6 and IL-8 enhanced cell invasion as measured using a Boyden chamber. While IL-6 enhanced cell invasion, our studies suggested that IL-8 may be a stronger inducer of cell invasion. However, we find that hypoxia induces an approximate 5–8 fold increase in IL-6 and IL-8 production in RCC cells at 48 hours; suggesting that both interleukins play a role in RCC cell invasion. The molecular aspects of cell invasion are complex and involve multi-step processes requiring proteolysis of matrix components and cell migration. The biological roles by which individual IL-6 and IL-8 mediate these complex processes are under current investigation. The role of Nox4-derived ROS in mediating IL-6 and IL-8-dependent RCC cell invasion is supported by our findings that pretreatment of RCC cells with the antioxidant, NAC or the Nox inhibitor, DPI in addition to specific Nox4 silencing with siRNA reduces IL-6 and IL-8 production and this conditioned media is unable to stimulate RCC cell invasion when compared to hypoxia conditioned media. Clinically relevant is the fact that the AMPK activator, AICAR, which we show in this study to reduce Nox4 expression and IL-6 and IL-8 production, reduces RCC cell invasion *in vitro* and in *ex vivo* RCC cells isolated from a human clear cell carcinoma tumor.

Our observations indicate that pharmacological activation of AMPK and antioxidants targeting Nox4 may represent a relevant therapeutic intervention to reduce IL-6- and IL-8-induced inflammation and cell invasion in RCC.

## Supporting Information

Figure S1 **Effects of** **hypoxia on the production of inflammatory markers in RCC 786-O cells.** A) CD40-L B) INF-gamma C) Interleukin-1A D) Interleukin-1B E) Interleukin-17 and F) TNF-alpha secretion by RCC 786-O exposed to normoxic (norm) or hypoxic conditions for short (T15-180min) or long (T24-T72hr) time points was determined by mosaic ELISA as outlined in materials and methods.
(TIF)

Figure S2 **Effects of** **hypoxia on the production of inflammatory markers in RCC4 cells.** A) CD40-L B) INF-gamma C) Interleukin-1A D) Interleukin-1B E) Interleukin-17 and F) TNF-alpha secretion by RCC 786-O exposed to normoxic (norm) or hypoxic conditions for short (T15-180min) or long (T24-T72hr) time points was determined by mosaic ELISA as outlined in materials and methods.
(TIF)
