# Supplementary figures and images for: Nox4 Mediates Renal Cell Carcinoma Cell Invasion through Hypoxia-Induced Interleukin 6- and 8- Production
Source: PLoS One. 2012 Jan 27;7(1):e30712. doi: 10.1371/journal.pone.0030712 (PMC3267761; doi:10.1371/journal.pone.0030712)

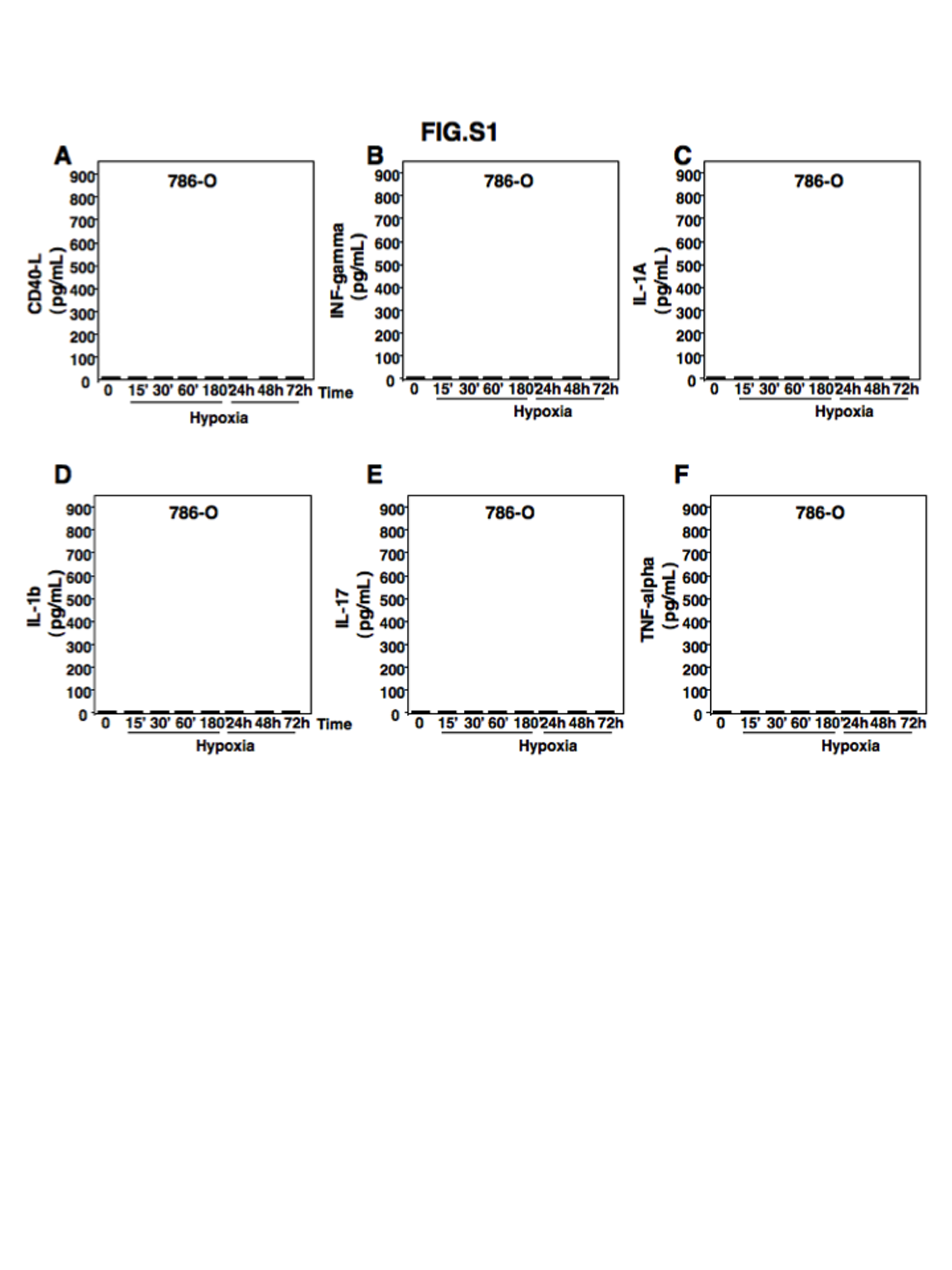

Supplement: Figure S1 — Effects of hypoxia on the production of inflammatory markers in RCC 786-O cells. A) CD40-L B) INF-gamma C) Interleukin-1A D) Interleukin-1B E) Interleukin-17 and F) TNF-alpha secretion by RCC 786-O exposed to normoxic (norm) or hypoxic conditions for short (T15-180min) or long (T24-T72hr) time points was determined by mosaic ELISA as outlined in materials and methods. (TIF) [file pone.0030712.s001.tif]

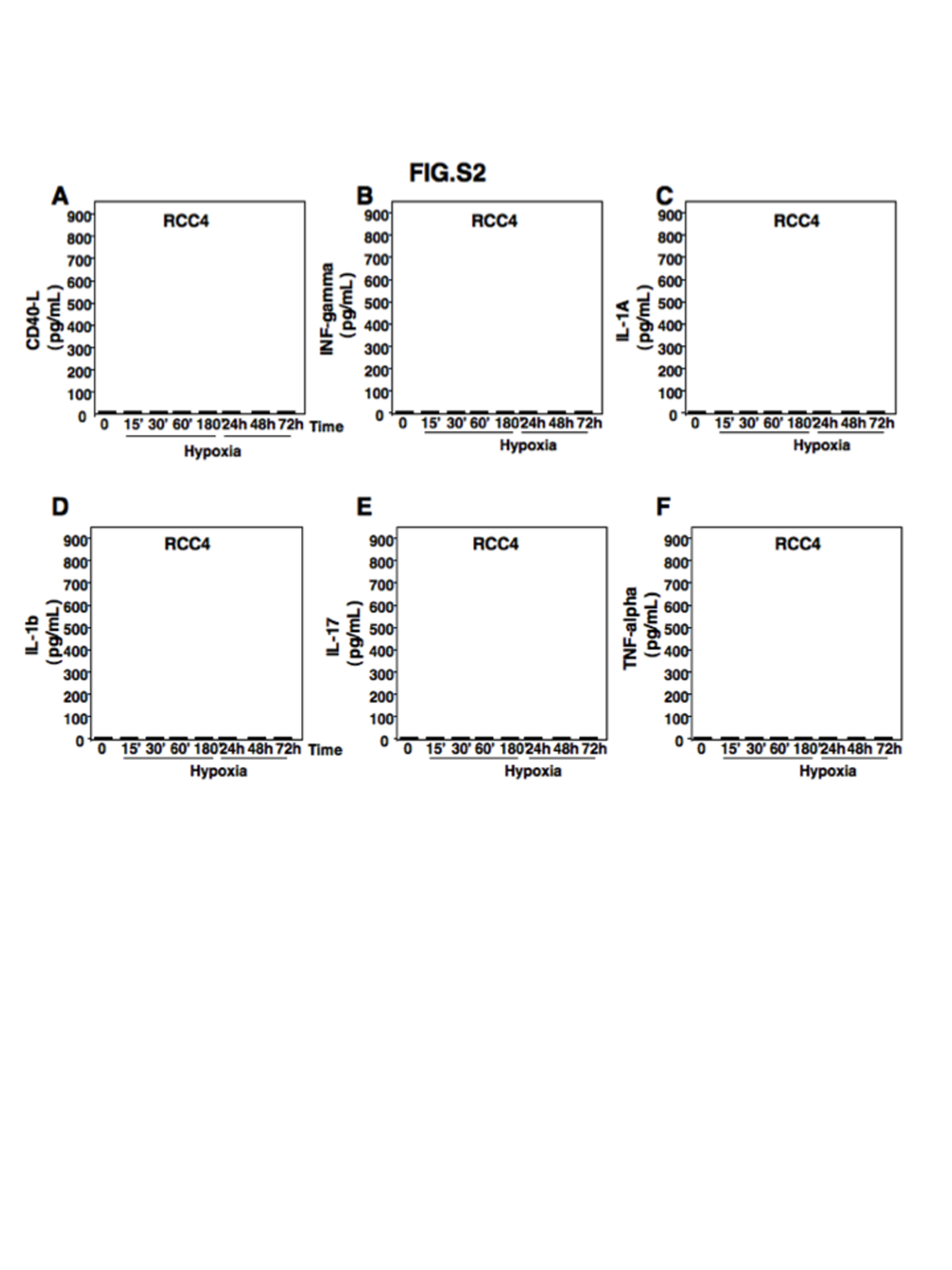

Supplement: Figure S2 — Effects of hypoxia on the production of inflammatory markers in RCC4 cells. A) CD40-L B) INF-gamma C) Interleukin-1A D) Interleukin-1B E) Interleukin-17 and F) TNF-alpha secretion by RCC 786-O exposed to normoxic (norm) or hypoxic conditions for short (T15-180min) or long (T24-T72hr) time points was determined by mosaic ELISA as outlined in materials and methods. (TIF) [file pone.0030712.s002.tif]
